# Prevalence of Self-Reported Chronic Non-Communicable Diseases among Adults in Addis Health and Demographic Surveillance System (Addis-HDSS), Addis Ababa, Ethiopia

**DOI:** 10.4314/ejhs.v34i2.9S

**Published:** 2024-12

**Authors:** Semira Abdelmenan, Meaza Demissie, Elsabet Wujira, Sitota Tsegaye, Hanna Gulema, Hanna Yemane Berhane, Gadise Bekele, Nebiyou Fasil, Dongqing Wang, Wafaie Fawzi, Alemayehu Worku, Yemane Berhane

**Affiliations:** 1 Department of Epidemiology and Biostatistics, Addis Continental Institute of Public Health, Addis Ababa, Ethiopia; 2 Department of Global Health and Health Policy, Addis Continental Institute of Public Health, Addis Ababa, Ethiopia; 3 Department of Reproductive Health and Population, Addis Continental Institute of Public Health, Addis Ababa, Ethiopia; 4 Department of Nutrition and Behavioral Science, Addis Continental Institute of Public Health, Addis Ababa, Ethiopia; 5 Department of Global and Community Health, College of Public Health, George Mason University, Fairfax, Virginia, United States of America; 6 Department of Global Health and Population, Harvard T.H. Chan School of Public Health, Harvard University, Boston, Massachusetts, United States of America

**Keywords:** Addis-HDSS, HDSS, NCDs, Prevalence, Chronic non-communicable diseases, Addis Ababa, Ethiopia

## Abstract

**Background:**

Chronic non-communicable diseases (NCDs) are a global health challenge, causing millions of deaths annually and contributing significantly to the global disease burden. Despite their prevalence in low- and middle-income countries (LMICs), NCDs receive limited global health financing. Ethiopia, like other LMICs, is experiencing a rising burden of NCDs. This study aimed to assess the self-reported prevalence of chronic NCDs and identify associated sociodemographic factors.

**Methods:**

A population-based cross-sectional study was conducted at the Addis Health Demographic Surveillance System (Addis-HDSS) site in Addis Ababa, Ethiopia. All adults (≥18 years) living in the Addis-HDSS sites were included. Data were collected using a structured electronic questionnaire on self-reported NCDs and sociodemographic variables. Binomial regression model was used to identify sociodemographic factors associated with self-reported NCDs.

**Results:**

Overall, 11.5% (95% CI: 11.3%-11.7%) of adults reported at least one NCD. The most prevalent conditions were hypertension (5.9%; 95% CI: 5.7%-6.1%) and diabetes mellitus (3.4%; 95% CI: 3.3%-3.5%). Older age (Adjusted Incidence Rate Ratio (AIRR): 5.47; 95% CI: 5.17-5.79), no formal education (AIRR: 1.58; 95% CI: 1.45-1.72), being formerly married (AIRR: 2.68; 95% CI: 2.47-2.91), and higher wealth quintiles (AOR: 1.16; 95% CI: 1.07-1.26) were statistically significant risk factors associated with NCDs.

**Conclusion:**

This study highlights the high burden of chronic NCDs among adults in Addis Ababa. The findings highlight the importance of addressing NCDs as a significant public health challenge. Expanding access to early prevention, diagnosis, and care is critical in urban settings.

## Introduction

Chronic non-communicable diseases (NCDs) are the leading causes of death worldwide, killing 41 million people each year, accounting for 74% of all adult deaths (1) and contributing to more than half of the global disease burden (2,3). NCDs—including cardiovascular diseases, diabetes, chronic respiratory diseases, and cancer—are often long-term conditions that develop gradually, resulting in years of disability and a lower quality of life (1).

In recent decades, the prevalence of NCDs has continued to rise in low- and middle-income countries (LMICs) (1,4). The burden of NCDs is expected to rise due to population aging, rapid urbanization, and changes in lifestyle and dietary habit (1,5), which are particularly pronounced in LMICs (1,6). Yet NCDs receive only 1–2% of global financing investment for health (6,7). According to the 2022 World Health Organization Report on World Health Statistics, the impact of NCDs is highest for LMICs where communicable diseases are still widely a public health concern (8). The disease burden and mortality linked to NCDs in Ethiopia have been on the rise, with minimal programmatic efforts (8–10), where cardiovascular diseases stand out as the most prevalent among all NCDs (11,12).

The underlying causes of NCDs are complex (10,13) including genetic predisposition, infections,, behavioral and biological, environmental, and sociodemographic factors (10). The sociodemographic factors that are associated with NCDs in Ethiopia from previous literature include older adults, females, people with lower levels of education and income, and people who live in urban areas (14–17).

Ethiopia implemented a national strategic action plan for the prevention and control of noncommunicable diseases in 2020 (10). In that effort, the limited evidence was a glaring shortcoming. Most research on NCDs among adults in Ethiopia was facility-based, which does not accurately reflect the burden of the diseases at a population level which is essential to develop context-appropriate interventions (18). Hence, the present study aimed to assess the Self-reported prevalence of NCDs, and the associated sociodemographic factors in the Addis Yeka Urban Health and Demographic Surveillance System site (Addis-HDSS), in Addis Ababa, Ethiopia.

## Methods

**Figure 1:**
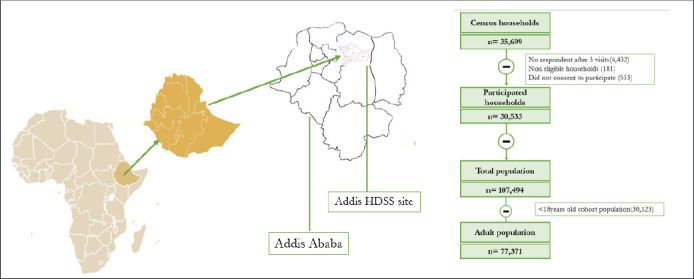
Study settings and flowchart of the participants in Addis HDSS, Addis Ababa, Ethiopia

**Study design and population**: This population-based cross-sectional study used data from the Addis-HDSS, in Addis Ababa. All permanent residents at the Addis-HDSS site were eligible to participate in the study. The census counted 107,494 individuals in the participating households; of which 77,371 were adult members of the households (Figure 1). Individuals who were 18 and above years old at the time of the survey were considered adults for this study.

**Data collection:** Data on self-reported NCDs and relevant sociodemographic variables were collected using a structured electronic questionnaire designed with the Open Data Kit (ODK) application (19). The outcome variable was the self-reported chronic NCDs. The question asked whether adult members of the household have any chronic NCDs including hypertension, diabetes mellitus, asthma, cancer, and other chronic illnesses. The heads of households or another adult member of the household were interviewed to provide information on behalf of all adults living in the household. The data collectors and supervisors were trained on survey procedures, questionnaires, research ethics, and related issues. A pretest was done in a similar setting which is in the Yeka sub-city namely Woreda 10.

**Statistical analysis**: Individuals were labeled as having any NCDs if they had at least one of the chronic conditions asked about. Sociodemographic variables considered for analysis include sex, age, marital status, education, employment status, and wealth status. Age was categorized into two groups: 18-39 and 40 and above; educational status was categorized into four groups: no formal education (those who do not have any formal schooling), primary education (those who completed grades 1-8), secondary education (those who completed grade 9-12), and higher education (those who have college-level education either vocational or technical). Marital status was categorized into three groups: never married, currently married, and formerly married (widowed, separated and divorced). Employment status was categorized into four groups: student, not employed, employed, and retired. Wealth status was classified into five groups: lowest, second, middle, fourth, and highest after computing the wealth index from multiple variables using principal component analysis.

A descriptive analysis was conducted to examine the sociodemographic variables and to assess the prevalence of NCDs. Categorical variables were summarized using frequency and percentage while continuous variables were presented using mean and standard deviation. Chi-square test was used to examine the relationship between explanatory and outcome variables. Both bivariate and multivariable analyses were performed to explore the crude and adjusted association between sociodemographic variables and the count of NCD occurrences, which ranged from 0 to 5.

Given the count nature of the outcome variable and its overdispersion compared to a Poisson distribution, a negative binomial regression model was employed. The goodness of fit was assessed using the likelihood ratio test. Multicollinearity was checked using a correlation matrix. The final model included the following sociodemographic variables: sex, age, marital status, educational status, and wealth status. Results of the negative binomial logistic regression analysis were reported in terms of incidence rate ratios (IRRs) and corresponding 95% confidence intervals (CIs). P-values of < 0.05 were considered statistically significant. Analysis was conducted using the Statistical software program Stata version 17.0 (20).

**Ethical considerations**: Ethical approval was obtained from the Ethics Review Committee of Addis Continental Institute of Public Health. Written informed consent was obtained from each participating household, and data were anonymized to ensure confidentiality.

## Results

A total of 77,371 adults participated in the study. The average age was 37.6 years (SD: 15), and more than half (56.7%) of the participants were female. About forty-six percent (46.5%) of the study participants were currently married/in union. One-third (34.0%) of the participants had higher education and the majority (59.9%) of them were employed (Table 1).

**Table 1:** Demographic characteristics of the participants in the Addis HDSS, Addis Ababa, Ethiopia

Characteristics (N=77,371)		n (%)/mean (SD)
Sex	Male	33,485 (43.3%)
	Female	43,886 (56.7%)
Age in years		37.6 (15.5)
Age group in years	18-39	49,948 (64.5%)
	>=40	27,453 (35.5%)
Marital status	Never married	30,456 (39.4%)
	Formerly married	10,912 (14.1%)
	Currently married	36,003 (46.5%)
Educational status	No formal education	7,044 (9.1%)
	Primary education	17,565 (22.7%)
	Secondary education	26,441 (34.2%)
	Higher education	26,321 (34.0%)
Employment status	Student	7,213 (9.3%)
	Not employed	19,820 (25.6%)
	Employed	46,351 (59.9%)
	Retired	3,987 (5.2%)
Wealth quintile	Lowest	13,758 (17.8%)
	Second	12,755 (16.5%)
	Middle	14,702 (19.0%)
	Fourth	17,294 (22.4%)
	Highest	18,862 (24.4%)

The prevalence of self-reported NCDs was 11.5% (95% CI: 11.3%-11.7%). The most prevalent reported NCDs were hypertension (5.9%; 95% CI: 5.7%-6.1%), diabetes mellitus (3.4%; 95% CI: 3.3%-3.5%), and asthma 1.5% (95% CI: 1.4%-1.6%) (Table 2).

**Table 2:** Prevalence of self-reported non-communicable diseases among the adult population of the Addis HDSS, Addis Ababa, Ethiopia (N=77,371)

NCD	Prevalence (%)	95% CI	
Any NCD	11.51	11.29	11.74
Hypertension	5.89	5.72	6.06
Diabetes mellitus	3.38	3.25	3.51
Asthma	1.51	1.42	1.60
HIV/AIDS	0.33	0.29	0.37
Gastric diseases	0.41	0.37	0.46
Kidney diseases	0.29	0.26	0.33
Heart diseases	0.37	0.33	0.42
Cancer	0.09	0.07	0.11
All other chronic illnesses*	2.04	1.94	2.14

* All other chronic illness includes chronic respiratory diseases, epilepsy, mental illness, liver diseases, gout arthritis, and Dementia/Alzheimer's.

The prevalence of NCDs varied by sociodemographic factors. The prevalence was higher among females (12.2%) compared to males (10.6%), in those aged 40+ years (26.0%) compared to those aged 18-39 years (3.5%), and among those with no formal education (26.4%). There was also an increasing trend in prevalence with higher wealth quintiles (Table 3). The prevalence of NCD multimorbidity (two or more NCDs) was 11.2%, with higher rates among older adults and those in higher wealth categories (Table 4).

**Table 3:** Distribution of self-reported non-communicable diseases among the adult population of the Addis HDSS, Addis Ababa, Ethiopia

Sociodemographic status		Prevalence (%)	95% CI	
			
			Lower bound	Upper Bound
Sex	Male	10.64	10.31	10.97
	Female	12.18	11.88	12.49
Age	18-39	3.53	3.37	3.70
	>=40	26.03	25.51	26.55
Marital status	Never married	3.57	3.37	3.79
	Formerly married	29.01	28.17	29.87
	Currently married	12.93	12.58	13.28
Employment status	Student	1.75	1.47	2.08
	Not employed	16.23	15.72	16.75
	Employed	7.91	7.66	8.16
	Retired	47.65	46.11	49.21
Educational status	No formal education	26.39	25.37	27.43
	Primary education	14.81	14.30	15.35
	Secondary education	8.79	8.46	9.14
	Higher education	8.06	7.74	8.40
Wealth quintile	Lowest	9.52	9.04	10.02
	Second	10.88	10.35	11.43
	Middle	10.99	10.50	11.51
	Fourth	12.25	11.77	12.75
	Highest	13.12	12.65	13.61

**Table 4:** Association between the number of self-reported chronic NCDs and sociodemographic factors among adults in Addis-HDSS, 2023

Sociodemographic status	Number of self-reported chronic NCDs Percent (95% CI)		

	0	1	>=2
Overall	88.49 (88.26, 88.71)	0.28 (0.25, 0.32)	11.23 (11.01, 11.45)
Sex			
Male	89.36 (89.03, 89.69)	0.16 (0.12, 0.21)	10.48 (10.15, 10.81)
Female	87.82 (87.51, 88.12)	0.38 (0.32, 0.44)	11.80 (11.50, 12.11)
Age			
18-39	96.47 (96.30, 96.63)	0.12 (0.12, 0.16)	3.41 (3.25, 3.57)
≥40	73.97 (73.45, 74.49)	0.58 (0.49, 0.67)	25.45 (24.94, 25.97)
Marital status			
Never married	29,368 (96.43)	42 (0.14)	940 (3.09)
Formerly married	7,746 (70.99)	109 (1.00)	2,247 (20.59)
Currently married	31,349 (87.07)	69 (0.19)	3,630 (10.08)
Employment status			
Student	98.25 (97.92,98.53	0.10 (0.05,0.20)	1.65 (1.38,1.97)
Not employed	83.77 (83.25,84.28)	0.43 (0.35,0.54)	15.80 (15.30,16.31)
Employed	92.09 (91.84,92.34)	0.26 (0.22,0.31)	7.65 (7.41,7.89)
Retired	52.35 (50.79,53.89)	0.18 (0.08,0.37)	47.48 (45.93,49.03)
Educational status			
No formal education	73.61 (72.57, 74.63)	0.88 (0.69, 1.13)	25.51 (24.51, 26.54)
Primary school	85.19 (84.65, 85.70)	0.51 (0.41, 0.62)	14.31 (13.80, 14.83)
Secondary school	91.21 (90.86, 91.54)	0.21 (0.16, 0.27)	8.59 (8.25, 8.93)
Higher education	91.94 (91.60, 92.26)	0.05 (0.03, 0.09)	8.01 (7.69, 8.34)
Wealth status			
Lowest	90.48 (89.98, 90.96)	0.55 (0.44, 0.69)	8.97 (8.50, 9.46)
Second	89.12 (88.57, 89.65)	0.46 (0.36, 0.60)	10.42 (9.90, 10.96)
Middle	89.01 (88.49, 89.50)	0.29 (0.21, 0.39)	10.71 (11.58, 11.22)
Fourth	87.75 (87.25, 88.23)	0.19 (0.14, 0.27)	12.06 (8.89, 12.56)
Highest	86.88 (86.39, 87.35)	0.05 (0.03, 0.10)	13.07 (12.60, 13.56)

After adjusting for sociodemographic factors, the multivariable negative binomial regression model showed that increasing age, being formerly or currently married, having no education, and highest wealth status were associated with self-reported NCDs. After adjusting for other variables, participants aged 40 years and above had a significantly higher incidence rate compared to those aged 18-39 (Adjusted IRR: 5.47, 95% CI: 5.17-5.79, p < 0.001). Formerly, and currently married individuals also showed significantly higher rates (Adjusted IRRs: 2.68 and 1.93, respectively, p < 0.001). Lower education levels were linked with higher incidence rates, with adjusted IRRs decreasing as education increased. Wealthier participants, particularly in the middle, fourth, and highest wealth quintiles, showed significantly higher rates compared to the lowest quintile (Table 5).

**Table 5:** Factors associated with self-reported chronic NCDs among adults in Addis-HDSS, 2023

Factors	Crude IRR (95% CI)	P value	Adjusted IRR (95% CI)	P value
Sex				
Male	ref		ref	
Female	1.14 (1.08,1.20)	<0.001	1.05 (0.99,1.11)	0.067
Age				
18-39	ref		ref	
>=40	8.18 (7.80,8.60)	<0.001	5.47 (5.17, 5.79)	<0.001
Marital status				
Never married	ref		ref	
Formerly married	8.89 (8.24,9.58)	<0.001	2.68 (2.47,2.91)	<0.001
Currently married	3.89 (3.65, 4.13)	<0.001	1.93 (1.81,2.06)	<0.001
Educational status				
No formal education	3.31 (3.03, 3.62)	<0.001	1.58 (1.45,1.72)	<0.001
Primary school	1.84 (1.71,1.97)	<0.001	1.47 (1.37,1.57)	<0.001
Secondary school	1.08 (1.01, 1.15)	0.022	1.12 (1.05,1.19)	<0.001
Higher education	ref		ref	
Wealth status				
Lowest	ref		ref	
Second	1.16 (1.06,1.26)	0.002	1.06 (0.97,1.15)	0.191
Middle	1.20 (1.10,1.32)	<0.001	1.08 (1.00, 1.18)	0.045
Fourth	1.36 (1.25,1.48)	<0.001	1.10 (1.02,1.19)	0.014
Highest	1.48 (1.37,1.61)	<0.001	1.16 (1.07,1.26)	<0.001

## Discussion

This study found a self-reported NCD prevalence of 11.5%, with hypertension and diabetes mellitus being the most common conditions. The study identified significant sociodemographic factors associated with NCDs, including age, marital status, educational level, and wealth. Older adults, those with no formal education, and those in higher wealth quintiles were more likely to report NCDs.

Many studies across the globe have consistently reported a high burden of NCDs including in LMICs (2,21). The prevalence of NCDs in this study was lower than what was reported from a meta-analysis of community-based studies conducted in Ethiopia (29-35%) (12), and the African region NCDs burden which was reported as approximately closer to 30% in 2019 by African Center for Disease Control and Prevention (22). The difference might have resulted as the studies included in the meta-analysis include studies conducted in specific high-risk groups like truck drivers (23), and studies used mortality as an indicator of NCDs burden based on physician review of verbal autopsy to identify the probable cause of death (24,25). Additionally, age differences in the study population may also contribute to the disagreement; this study included study participants 18 years of age and above while most studies and national NCD mortality data focused only on the older population. On the contrary, the prevalence of NCDs in this study was higher than in the studies conducted in Dabat HDSS in northern Ethiopia 1.7% (26), and a population-based survey of NCDs in Southwest Ethiopia 8.9% (27). The high prevalence of self-reported NCDs observed in this study might have resulted from a place of residence. This study was conducted in a predominantly urban area while the other studies were conducted in relatively rural areas in which the population is more physically active than the urban residents.

The prevalence of NCD multimorbidity was one in ten among all participants, rising to one in four among those aged 40 and above. This result is similar to global NCD burden reports (6) and with previous research in Ethiopia (9,12,28) which indicate large proportion of individuals suffer from comorbidities. Consistent with our findings, the two most common NCDs were diabetes mellitus and hypertension, both of which are extremely widespread illnesses worldwide that impact a sizable portion of the population. These illnesses often coexist with other health problems and are relatively easier to diagnose compared to some other chronic conditions. Blood pressure measurements, and blood glucose tests are not expensive and can be done at home.

The study also revealed a slight difference in NCD prevalence between genders, with females showing a marginally higher prevalence. However, this difference was attenuated after adjusting for other sociodemographic factors.

Age was a significant factor, with individuals aged 40 and above being six times more likely to report NCDs than those aged 18-39 years. This is consistent with global and local (5,12) findings, as the risk of NCDs increases with age due to accumulating risk factors and decreased physical activity (29–31).

Marital status also influenced NCD prevalence, with formerly or currently married individuals having higher rates of NCDs compared to never-married individuals. Similar findings were observed in other parts of Ethiopia (31) and elsewhere (32–34). The available evidence indicated possible explanations for the effect of marital status on NCDs. Some other behavioral, cultural, and societal factors that were not measured in this and other similar studies could account for the observed association. Possibly, cultural factors like social norms and support systems, psychological factors like emotional stress and depression, and socioeconomic burden accumulating to formerly married individuals may explain the association between being formerly married with having NCDs (31–33). Moreover, married individuals specifically women may have other unmeasured factors like utilization of hormonal contraceptives that are a risk factor for NCDs (32). However, further studies on how marital status is associated with NCD are required to explain these findings.

Educational status had an inverse relationship with NCD prevalence, with those having no formal education being three times more likely to report NCDs than those with higher education. This aligns with previous studies (31,35,36), suggesting that higher education may lead to better health literacy (37,38) and healthier behaviors (39,40).

The increased prevalence of NCDs in the highest wealth quintile as compared to the lowest quintile is consistent with studies from other LMICs (41). However, it is important to note that the relationship between wealth and NCDs is complex and many factors contribute to this relationship. In general, studies from LMICs showed the likelihood of NCDs increase among people with high socio-economic status whereas, for those from high-income countries, the relationship is the inverse (42,43). In LMICs, there is a nutritional transition that incorporates changes in dietary habits and lifestyle. People in higher wealth quintiles from LMICs tend to eat more processed foods, which is perceived as a sign of modernization (44). In LMICs, high-calorie foods, especially from animal sources are costly and accessible mainly by the people in the higher wealth quintile (45,46). In contrast, diets of individuals with high socio-economic status in high-income countries tend to be of better quality leading to the likelihood of NCDs to be lower (43).

There are potential strengths and limitations related to the findings of this study. This study is the largest community survey in Addis Ababa on the prevalence of NCDs with 77,371 participants. Our findings generate evidence on the prevalence of NCDs in the capital city and identify sociodemographic factors associated with NCDs. The main limitation of this study is that NCD prevalence was assessed based on self-report. Therefore, the use of self-reporting to assess NCD prevalence likely led to the underestimation of the prevalence of NCDs in the Addis-HDSS site. However, by developing a correction factor in future studies a self-reported prevalence can be a useful indicator to monitor the trend and for planning interventions. Additionally, incorporating laboratory tests and comprehensive clinical assessments to validate self-reported prevalence data will be done in future follow up studies.

In conclusion, the prevalence of self-reported chronic NCDs among the adult population in the Addis-HDSS site was high, considering the majority of the population studied is relatively young. Appropriate actions need to be taken to strengthen promotive, preventive, curative, and rehabilitative services in the city. The actions should focus on rising awareness on NCDs, screening for NCDs, strengthening the health system to accommodate the high burden of NCDs, and further research to better understand the NCDs burden and develop new and effective interventions.
